# Prognosis for long-term survival and renal recovery in critically ill patients with severe acute renal failure: a population-based study

**DOI:** 10.1186/cc3879

**Published:** 2005-10-25

**Authors:** Sean M Bagshaw, Kevin B Laupland, Christopher J Doig, Garth Mortis, Gordon H Fick, Melissa Mucenski, Tomas Godinez-Luna, Lawrence W Svenson, Tom Rosenal

**Affiliations:** 1 Fellow, Departments of Critical Care Medicine, and Community Health Sciences, Calgary Health Region and University of Calgary, Calgary Alberta, Canada; 2 Assistant Professor, Departments of Critical Care Medicine, Medicine, Community Health Sciences and Pathology and Laboratory Medicine, Calgary Health Region and University of Calgary, Calgary Alberta, Canada; 3 Associate Professor, Departments of Critical Care Medicine, Medicine and Community Health Sciences, Calgary Health Region and University of Calgary, Calgary Alberta, Canada; 4 Clinical Assistant Professor, Department of Medicine, Calgary Health Region and University of Calgary, Calgary Alberta, Canada; 5 Professor, Department of Community Health Sciences, Calgary Health Region and University of Calgary, Calgary Alberta, Canada; 6 Research Assistant, Department of Medicine, Calgary Health Region and University of Calgary, Calgary Alberta, Canada; 7 Clinical Assistant Professor, Departments of Critical Care Medicine and Medicine, Calgary Health Region and University of Calgary, Calgary Alberta, Canada; 8 Associate Professor, Department of Community Health Sciences, Calgary Health Region and University of Calgary, Calgary Alberta, Canada; 9 Epidemiologist, Health Surveillance Branch, Alberta Health and Wellness, Edmonton Alberta, Canada; 10 Associate Professor, Department of Public Health Sciences, University of Alberta, Edmonton Alberta, Canada; 11 Associate Professor, Departments of Medicine and Community Health Sciences, Calgary Health Region and University of Calgary, Calgary Alberta, Canada

## Abstract

**Introduction:**

Severe acute renal failure (sARF) is associated with considerable morbidity, mortality and use of healthcare resources; however, its precise epidemiology and long-term outcomes have not been well described in a non-specified population.

**Methods:**

Population-based surveillance was conducted among all adult residents of the Calgary Health Region (population 1 million) admitted to multidisciplinary and cardiovascular surgical intensive care units between May 1 1999 and April 30 2002. Clinical records were reviewed and outcome at 1 year was assessed.

**Results:**

sARF occurred in 240 patients (11.0 per 100,000 population/year). Rates were highest in males and older patients (≥65 years of age). Risk factors for development of sARF included previous heart disease, stroke, pulmonary disease, diabetes mellitus, cancer, connective tissue disease, chronic renal dysfunction, and alcoholism. The annual mortality rate was 7.3 per 100,000 population with rates highest in males and those ≥65 years. The 28-day, 90-day, and 1-year case-fatality rates were 51%, 60%, and 64%, respectively. Increased Charlson co-morbidity index, presence of liver disease, higher APACHE II score, septic shock, and need for continuous renal replacement therapy were independently associated with death at 1 year. Renal recovery occurred in 78% (68/87) of survivors at 1 year.

**Conclusion:**

sARF is common and males, older patients, and those with underlying medical conditions are at greatest risk. Although the majority of patients with sARF will die, most survivors will become independent from renal replacement therapy within a year.

## Introduction

Severe acute renal failure (sARF) in the critically ill patient is associated with high rates of morbidity, mortality, and consumption of healthcare resources [1-6]. Population-based studies conducted in Australia and Europe have estimated the annual incidence of sARF at 4.2 to 13.4 per 100,000 population; however, these studies were not designed to assess risk factors, long-term survival and renal recovery outcomes [5,7-9]. Although several hospital-based cohort studies have suggested that several factors, including increasing age, sepsis syndrome, and cardiovascular or pulmonary organ failure, increase the risk for developing sARF, these studies potentially suffer from selection bias, and no study to date has been adequately designed to determine actual risk factors in a non-specific general population [2,10-13]. Population-based studies have identified in-hospital case-fatality rates for sARF ranging between 45% and 70% [5,7-9]. Although renal recovery is reported to occur in many patients surviving sARF, this rate is not well described because of inadequate duration or consistency of follow-up [3-12,14-21]. As a result, the long-term survival and renal recovery outcomes for sARF are currently unknown.

An understanding of the epidemiology of sARF is important to establish its overall burden and risk factors for development and for surveillance or devising potential preventive strategies. Furthermore, knowledge of the outcome of sARF is important to aid clinicians, patients, and their families in decision-making regarding patient management choices in the intensive care unit (ICU). The objectives of this study were, therefore, to define the incidence of and quantify the risk factors for developing sARF, and to establish the long-term outcome and its determinants in a large well-defined population.

## Materials and methods

### Patient population

The Calgary Health Region (CHR) provides virtually all hospital care to the residents of the cities of Calgary and Airdrie and approximately 20 nearby towns and villages (2001 adult population 728,207) [22]. Adult critically ill patients in the CHR are managed in closed ICUs by dedicated intensivists under the direction of the Department of Critical Care Medicine, University of Calgary and the CHR. The study population consisted of all adult (≥18 years) residents of the CHR admitted to any of the three multidisciplinary ICUs or the cardiovascular surgery ICU from May 1 1999 to April 30 2002. The study protocol was approved by the Conjoint Health Research Ethics Board at the University of Calgary and CHR prior to commencement.

### Study protocol

The study used a population-based surveillance cohort design. The ICU Tracer database, a clinical research and departmental support database that prospectively and routinely records data on all patients admitted to adult ICUs in the CHR was used to identify patients with sARF among all admissions to the study ICUs. The ICU Tracer database collected detailed clinical and physiologic data for all sARF patients on the dates of admission and initiation of renal replacement therapy (RRT). A trained research nurse and physician reviewed hospital medical records to obtain detailed clinical information using standardized data forms for all patients identified with sARF. Potential factors contributing to the development of sARF were considered if identified within 10 days preceding initiation of RRT.

### Study definitions

sARF was defined as the new requirement for RRT with evidence of renal dysfunction (serum creatinine ≥ 150 μmol/l) at the time of or during ICU admission [8,9]. Those patients that received RRT for any indication in the absence of renal dysfunction (i.e. toxin ingestion/overdose) or patients with end-stage renal disease already receiving chronic RRT or patients having their first RRT >48 h prior to ICU admission were excluded. Renal replacement therapy in the ICU encompassed continuous renal replacement therapy (CRRT) and/or intermittent hemodialysis (IHD). No patient received RRT in the form of peritoneal dialysis while admitted to ICU. The decision for initiation of RRT was made at the discretion of the attending intensivist. A regional protocol has been implemented to guide in the initial RRT prescription and method of anticoagulation for CRRT as previously described [23]. IHD was prescribed in consultation with the CHR clinical nephrology service. Chronic renal dysfunction was defined as a pre-existing serum creatinine ≥150 μmol/l for at least six months prior to ICU admission. Oliguria was defined as the production of <500 ml of urine in the 24 h preceding assessment. The definitions by Liano *et al*. [7] and clinical sensibility were used for classification of etiologies of sARF and characterization of indications for RRT upon review of available patient medial record data immediately preceding initiation of RRT. Pre-renal etiology was defined as directed therapy (i.e. volume repletion and/or increased cardiac output) being successful in improving and/or correcting renal function. Intra-renal etiology of sARF was defined when renal function failed to improve after correction for possible pre-renal etiologies and exclusion of hepatorenal syndrome and post-renal etiologies. Intra-renal etiology was further classified into acute tubular necrosis, acute glomerulo-nephritis, acute tubulo-interstitial nephritis or vascular etiology based on the presence of predisposing factors, histological evidence, serum or urinary markers and/or high clinical suspicion [7]. A study physician (SMB) reviewed all abstracted data forms prior to entry into the study database in order to ensure consistency of application of study definitions and diagnoses.

Severity of illness at ICU admission was assessed using the Acute Physiology and Chronic Health Evaluation (APACHE) II score [24]. The presence and evaluation of selected pre-existing co-morbidities was assessed using the Charlson Co-morbidity Index [25]. Shock preceding or at the time of initiation of RRT was defined as mean arterial pressure <70 mmHg and need for vasopressor therapy. The presence of sepsis, septic shock, and acute respiratory distress syndrome preceding or at the time of the initiation of RRT was defined according to consensus guidelines [26,27].

### Data sources

The size and demographic profile of the CHR adult population at risk during 1999 to 2002 were obtained by using population data from the Alberta Health Registry [22]. The prevalence of selected underlying chronic illnesses was estimated based on Canadian survey data [28,29], with the exception of the prevalence of chronic kidney disease, which was determined by United States survey data [30]. Long-term renal outcome and RRT dependence status was determined from the Southern Alberta Renal Program database that maintains information on all patients in southern Alberta on RRT [31]. The long-term mortality outcome status was obtained through linkage of the Death Registration Database maintained by Alberta Vital Statistics and the Alberta Health and Wellness registry. Alberta Health and Wellness maintains information on all residents of Alberta eligible for publicly funded healthcare coverage (>99% of the population is included in this registry). The use of both information systems ensured completeness of the linkage process. Data were exported from the source databases and linked using Access 2003 (Microsoft Corporation, Redmond, WA, USA).

### Statistical analysis

Analysis was performed using Stata version 8.2 (Stata Corporation, College Station, TX, USA). To avoid assessment of multiple outcomes for a single patient, only the first ICU presentation associated with sARF was analyzed for patients with multiple ICU admissions. Normally or near normally distributed variables were reported as means with standard deviations (SDs) and compared using Student's t-test. Non-normally distributed continuous data were reported as medians with inter-quartile ranges (IQRs) and compared using the Mann Whitney *U* test. Categorical data are compared using Fisher's Exact Test. Population-based incidence rates were calculated and reported as relative risks (RRs) with exact 95% confidence intervals (CIs) [32]. A multivariable logistic regression model was developed to assess factors in patients with sARF associated with mortality at 1 year. The initial model included selected variables known to potentially confound and/or modify the association of sARF and death at 1 year, including age, sex, Charlson co-morbidity index, APACHE II score, and admission type (medical versus surgical). The second model added variables to the first model if found to be significant at the *p* < 0.1 level in univariate analysis. Sequential elimination of variables was performed by the likelihood ratio method to develop the final parsimonious model. Appropriate diagnostic tests to ensure no violation of mathematical assumptions were performed. Model calibration and discrimination were assessed using the Hosmer-Lemeshow goodness-of-fit test (degrees of freedom (8)) and the area under the receiver operator characteristic (AuROC) curve, respectively. Results are reported as odds ratios (ORs) with 95% CI. The rate of renal recovery among patients surviving sARF was determined at 1-year follow-up and expressed as a proportion.

## Results

During the study period, 5,693 adult residents of the CHR had 6,762 admissions to a CHR ICU. Of these, 62% were male, the median (IQR) age was 64.9 (50.6 to 74.5) years and mean (±SD) APACHE II score was 24.9 ± 8.7 points at ICU admission. A total of 343 (6%) patients received RRT at least once during an ICU admission, of which 103 (1.8%) were excluded: 92 (1.6%) for outpatient chronic RRT, 6 for RRT in an ICU without critical illness and 5 received RRT for toxin removal in the absence of renal disfunction. Therefore, 240 (4.2%) patients were diagnosed with sARF for an overall annual incidence of 11.0 per 100,000 population. The incidence of sARF was stable over the three years of the study.

### Population-based risk factors and mortality for severe acute renal failure

**Figure 1:**
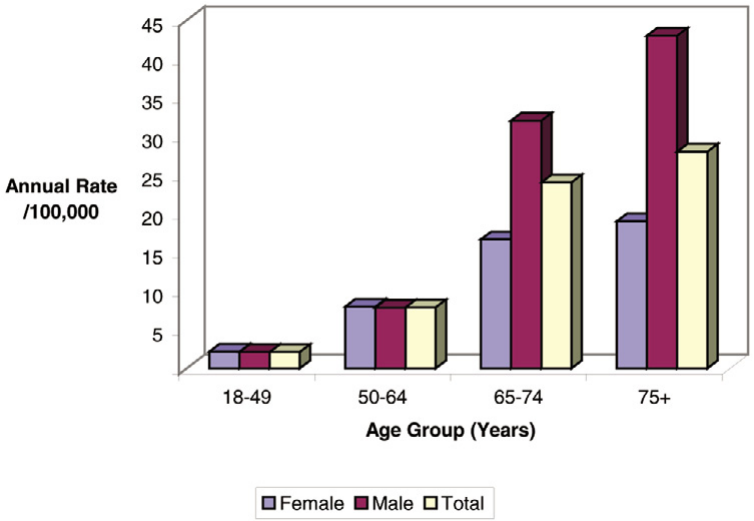
Age and sex-specific incidence rates of severe acute renal failure among adult residents admitted to a Calgary Health Region intensive care unit.

The annual incidence rate of sARF was higher in males compared to females (13.0 versus 9.1 per 100,000 population; RR 1.4; 95% CI, 1.1–1.9, *p* = 0.006). This relationship was more pronounced for those ≥65 years old with higher risk in males (70.0 versus 31.9 per 100,000 population; RR 2.2; 95% CI, 1.5–3.2, *p* < 0.0001) compared with no significant difference in risk between sexes for those <65 years old (6.4 versus 5.5 per 100,000 population; RR 1.2; 95% CI, 0.8–1.7, *p* = 0.44) (Figure 1).

Several groups were identified in the adult CHR general population as being at significantly higher risk for development of sARF, with patients with heart disease, stroke, and chronic lung disease at highest risk (Table 1).

**Figure 2:**
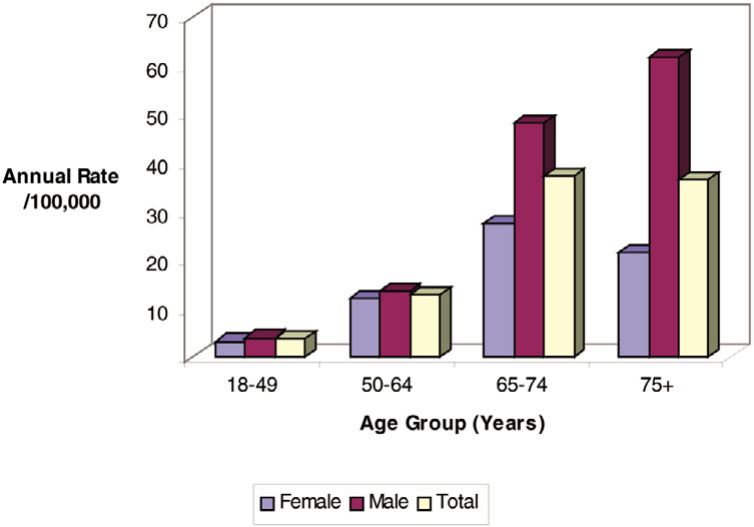
Age and sex-specific mortality rates of severe acute renal failure among adult residents admitted to a Calgary Health Region intensive care unit.

The population-based annual mortality rate associated with sARF was 7.3 deaths per 100,000 population. There was a trend toward a higher annual mortality rate in males compared with females (8.2 versus 6.3 per 100,000 population; RR 1.3; 95% CI, 0.9–1.8, *p* = 0.1); however, when further stratified by age ≥65 years, the annual mortality rate was significantly higher for males compared with females (47.5 versus 23.1 per 100,000 population; RR 2.1; 95% CI, 1.3–3.3, p < 0.001). There was no significant difference in mortality risk between sexes for age <65 years (3.6 versus 3.7 per 100,000 population; RR 0.99; 95% CI, 0.6–1.6, *p* = 0.96) (Figure 2).

### Clinical features and management

The majority (166/240, 69%) of diagnoses of sARF occurred within two days of ICU admission. The median (IQR) time to diagnosis of sARF was 4 (1 to 10.5) days after hospital admission and 1 (0 to 3) day after ICU admission. Among the 240 patients with sARF, 203 (85%) had intra-renal, 36 (15%) pre-renal, and one had a post-renal etiology (0.4%) (Table 2). Of the 203 patients with an intra-renal etiology, 180 (75%) had acute tubular necrosis, and 33 (14%) had vascular, 12 (5%) glomerular, and 9 (4%) interstitial etiologies. Toxic exposures included parenteral radiocontrast media in 107 (45%), aminoglycosides in 47 (20%) and amphotericin B in 10 (4%). The indications for institution of RRT were diuretic-resistant fluid overload in 178 (74%), metabolic acidosis in 87 (36%), uremia in 68 (28%), hyperkalemia in 59 (25%), and toxins in 4 (2%).

The modality of renal replacement in the ICU was exclusively CRRT in 147, exclusively IHD in 48, and some combination of regimens using both CRRT and IHD in 45 patients. A total of 941 and 343 patient-days of CRRT and IHD were performed, respectively. The median overall duration of RRT in the ICU was 3 (IQR; 1 to 9) days.

The overall median (IQR) ICU and hospital length of stay was 8.1 (3.4 to 16) and 22 (9 to 40) days, respectively. Of those who survived to hospital discharge, the median (IQR) ICU and hospital length of stay was 8.4 (3.6 to 19) and 37 (22 to 62) days, respectively.

### Long-term outcomes of severe acute renal failure

Among the 240 patients with sARF, 50% (n = 120) died during their ICU admission and 60% (n = 143) died prior to hospital discharge. The 28-day, 90-day, and 1-year case-fatality rates were 51% (n = 123), 60% (n = 143), and 64% (n = 153), respectively. Several categorical and continuous factors were associated with death at 1-year in univariate analysis, as shown in Tables 3 and 4. Factors not significantly associated with death at 1 year included sex, oliguria, etiology of sARF, or indication for RRT. A multivariable logistic regression model was developed to assess for independent factors associated with death at 1 year for patients with sARF and model variables are presented in Table 5. The model calibration and fit was excellent with an AuROC curve of 0.83 and a Hosmer-Lemeshow goodness-of-fit test (degrees of freedom (8)) result of *p* = 0.78.

Of patients with sARF who survived, 38% (46/120) and 68% (66/97) had recovered renal function to become RRT independent at ICU and hospital discharge, respectively. The rates of renal recovery in survivors at 28 and 90 days were 55% (64/117) and 71% (69/97), respectively. Of the 87 patients with sARF who survived to at least 1 year following admission to ICU, 78% (68/87) became independent of RRT after a median (IQR) duration of 11 (3 to 20) days while the remainder received chronic RRT. Of those requiring chronic RRT, 63% (12/19) had pre-existing chronic renal disfunction with a median (IQR) pre-admission serum creatinine of 232 (170 to 323) μmol/l. Thus, at 1 year following the diagnosis of sARF, only 28% (68/240) were alive and free of RRT. Compared to those that remained on chronic RRT, patients that recovered renal function were more likely to be male, non-diabetic, have a lower Charlson co-morbidity score, with a diagnosis of acute tubular necrosis and sepsis or septic shock.

## Discussion

This study describes the incidence and long-term mortality rates for critically ill patients with a diagnosis of sARF and the prognosis for long-term renal recovery in a well-defined non-specified population. The annual incidence of sARF in our well-defined population of 11.0 per 100,000 population per year is similar to previous studies from two other continents, 8.0 to 13.4 per 100,000 in Australia and 4.2 to 8.0 per 100,000 in Europe, respectively [5,7-9]. However, two of these studies are potentially prone to selection bias due to failure in clearly defining the geographic boundaries and classification of residency status of the study referral population for which the incidence of sARF was determined [8,33,34], and all these studies are limited as none were able to assess for risk factors, long-term survival or long-term renal recovery prognosis [8,9,33]. Recently, the multi-centre BEST study reported an estimated prevalence for ARF of 5.7% defined by the presence of oliguria and/or azotemia and 4.2% for sARF from 54 ICUs in 23 countries [21]. While the BEST study is the largest, most comprehensive completed to date and demonstrates a similar occurrence of sARF and in-hospital mortality with our study, it remains prone to selection bias and provided no long-term follow-up. Morgera *et al*. reported survival in a cohort of critically ill patients with sARF of 77% and 50% at 6 months and 5 years, respectively; however, this study is potentially prone to selection and information bias due to inclusion of patients receiving only CRRT and incomplete ascertainment of long-term survival status for the entire cohort [6]. This was unlikely to be a major source of bias in the present study because in the CHR, all critical care services are provided by ICUs included within this surveillance and the CHR is geographically isolated as a single provider of healthcare. Although we could have potentially missed sARF cases that developed in CHR residents while receiving medical attention not available in the CHR (i.e. cardiac, lung or liver transplantation) or while traveling abroad. Likewise, we could have potentially excluded sARF patients with peak serum creatinine <150 μmol/l, resulting in an under-estimation of the incidence of sARF. However, these sources of error, if present, are likely to be small and insignificant. Therefore, this study further establishes the major burden of disease in terms of occurrence, long-term mortality and renal prognosis attributable to sARF in a non-specified population.

Recent consensus recommendations for defining and categorizing acute renal failure have been presented; however, they have not yet been prospectively validated with long-term clinical outcomes such as mortality or renal recovery at 1 year [35]. This was published after completion of our study; thus, we used as our primary case-definition, acute renal failure severe enough, in the opinion of the treating intensivist, to warrant the initiation of RRT. This definition was selected because the initiation of RRT in critically ill patients has clinical relevance both in terms of severity of illness and for utilization of resources. Thus, a diagnosis of sARF and institution of RRT represents a considerable escalation in patient management. Further, we selected sARF due to simplicity in potentially generalizing our results across similar multi-disciplinary critically ill populations. One potential limitation of this case-definition is defining what factors contribute to the decision by the attending intensivist to initiate RRT, rather than specific indications for RRT. This was not addressed in our study and has yet to be prospectively studied. Another consideration is that, in general, there is likely to be heterogeneity across ICUs regarding who prescribes RRT (i.e. intensivist or nephrologist); however, in this regional critical care system, the decision to initiate RRT was made by the attending intensivist only.

A novel aspect of this study was that several selected underlying conditions were determined to be associated with an increased risk for development of sARF. While previous investigators have suggested that several factors, most notably increasing age, pre-existing renal insufficiency, co-morbid liver or cardiac disease, cancer, sepsis, and greater severity of illness are potential risk factors for sARF, no previous studies were designed to determine and quantify risk in a general population [2,10-13]. Although these estimates provide unbiased univariate population-based risk factors for development of sARF, one potential limitation is the complexity in interpretation without adjustment for potential confounders or effect modifiers. However, our study has shown that critically ill patients who were older, male, and have underlying co-morbid illnesses were at higher risk for developing sARF and may represent a future target population for surveillance, earlier intervention or preventive strategies.

Most studies of sARF in the critically ill population have focused on mortality and renal recovery at ICU and hospital discharge, therefore viewing sARF as an acute and short-term illness [3-5,8,9,11,18,21,36]. Assessment of outcomes at these points may underestimate the burden of disease attributable to sARF. Our data indicate that critically ill patients with sARF may remain ill with an increased risk for death for a duration greater than the total ICU or hospital length of stay. This has been similarly shown with sepsis and septic shock, where patients exhibit an increased risk of death following discharge from hospital [37-39]. Therefore, clearly defined long-term outcomes as demonstrated in our study, such as case-fatality at 1 year of 64% and rate of renal recovery in survivors of 78%, provide a more informative description of the morbidity and mortality attributable to sARF. Furthermore, the assessment of long-term outcome is important given the high cost associated with RRT in the ICU and continuing chronic RRT [1,4,5]. Likewise, independence from RRT is associated with improved overall quality of life and functional status [1]. Dependence on RRT at hospital discharge and at 90 days has been estimated to occur in 5% to 33% and 16% of patients, respectively [3-5,8,9,11,18,21,36,40]; however, this does not necessarily translate into long-term RRT dependence. Although the overall rate of dependence on RRT at hospital discharge in our study was comparably higher than other population-based studies, only 8% overall or 22% of survivors at 1 year remained on chronic RRT, an assessment duration more likely associated with permanent need for chronic RRT.

We identified five factors independently associated with death at 1 year (Table 5). Although the presence of co-morbid liver disease, higher admission APACHE II score, septic shock, and use of CRRT have been previously suggested, these studies are potentially biased due to the aforementioned limitations in assessment of mortality at ICU or hospital discharge [2,3,10,12-14,16-18,21,41,42]. An important variable included in this study not previously reported is the contribution of the Charlson co-morbidity index to the overall risk of death [25]. Previous hospital-based studies have included previous health status as an independent risk for death; however, this was generally assessed by use of the chronic health points component of the APACHE II score or the McCabe scale [10,16,41]

The need for CRRT was independently associated with death in our study after controlling for the confounding effects of co-morbid illness and disease severity. This is plausible considering that in our clinical practice CRRT is utilized in more unstable patients with great burden of illness, and a poorer expected outcome. Although similarly reported by Chertow *et al*. [3], this would appear to contradict several randomized studies suggesting no difference in mortality outcome between CRRT and IHD; however, these studies have methodological concerns, including failure of randomization, inadequate power to assess clinically meaningful differences in primary outcome and, importantly, did not assess the independent effect of dialysis modality on long-term outcomes such as mortality or renal recovery at 1 year [43-47].

In contrast to previous hospital-based studies and our pre-analysis prediction, none of older age, presence of pre-existing renal disease, need for mechanical ventilation or oliguria were independently associated with death [2,3,10,12-14,16,17,41,48].

The apparent lack of association of chronic renal insufficiency with death at 1 year in our study would appear counterintuitive considering the association of death and co-morbid illness. However, the presence of pre-existing renal disease in these patients likely afforded greater susceptibility to overt renal injury prompting RRT. Although not associated with death, sARF in patients with co-morbid renal disease may represent a cohort less likely to recover renal function and subsequently require chronic RRT as suggested by our study.

## Conclusion

We describe the first population-based study of sARF that documents the major burden of disease in terms of incidence, long-term mortality and prognosis of renal recovery in critically ill patients. Furthermore, our study identifies and quantifies the risk factors for acquisition of sARF and may serve as a rationale for surveillance or targeting preventive measures. Although we report that the case-fatality during the acute phase of sARF is very high, our data support that survivors of sARF have an excellent prognosis for long-term renal recovery. We believe this study represents an important contribution for physicians, patients, and their families and has the potential to greatly impact the care of critically ill patients by aiding in and providing well-informed overall management decisions.

## Key messages

• 	sARF is common in critically ill patients and males, older patients and those with pre-existing co-morbidities are at highest risk.

• 	A diagnosis of sARF is associated with high mortality and pre-existing co-morbidities, liver disease, higher APACHE II score upon ICU admission, septic shock and need for continuous renal replacement therapy are independently associated with long-term mortality.

• 	In those patients with severe acute renal failure who survive an episode of critical illness, the majority will recover renal function and become independent of renal replacement therapy by 1 year.

## Abbreviations

APACHE = Acute Physiology and Chronic Health Evaluation; AuROC = area under the receiver operator characteristic; CHR = Calgary Health Region; CI = confidence interval; CRRT = continuous renal replacement therapy; ICU = intensive care unit; IHD = intermittent hemodialysis; IQR = interquartile range; RR = relative risk; RRT = renal replacement therapy; sARF = severe acute renal failure; SD = standard deviation.

## Competing interests

The authors declare that they have no competing interests.

## Authors' contributions

SMB developed the study protocol, collected data, analyzed data, and wrote and revised the manuscript. KBL conceived the study, developed the study protocol, analyzed data, and provided critique of successive drafts of the manuscript. MM and GM collected data. LWS provided mortality outcome data. CJD, GM, GHF, TGL and TR participated in design of the study and provided critique of successive drafts of the manuscript. All authors read and approved the final manuscript.

## Figures and Tables

**Table 1:** Risk of sARF associated with selected co-morbidities among adult residents of the Calgary Health Region, Canada

Underlying condition	Number of patients with sARF (n = 240)	Estimated number with underlying condition at risk in CHR	Annual incidence (per 100,000 population)	Relative risk^a ^(Exact 95% CI)
Heart disease	120	85,576	140	24.0 (18.5–31.2)^b^
Stroke	44	21,394	206	22.0 (15.6–31.0)^b^
Chronic pulmonary disease	83	68,461	121	16.0 (12.1–21.0)^b^
Diabetes mellitus	72	85,576	84	10.3 (7.7–13.6)^b^
Cancer	38	42,788	89	9.2 (6.3–13.1)^b^
Connective tissue disease	12	21,394	56	5.2 (2.7–9.3)^b^
Chronic kidney disease	45	96,273	47	4.9 (3.5–6.8)^b^
Alcohol abuse	57	145,480	39	4.3 (3.1–5.8)^b^

^a^Relative risk calculated by ((Number of sARF patients with underlying condition/Number at-risk with underlying condition in CHR)/(Number of sARF patients without underlying condition/Number at-risk without underlying condition in CHR)). ^b^*p* value < 0.0001 for each underlying condition relative risk. Underlying conditions were defined by using the Charlson Co-morbidity Index [25]. The presence of alcohol abuse was defined by documentation in patient medical record or by history. CHR, Calgary Health Region; CI, confidence interval; sARF, severe acute renal failure.

**Table 2:** Summary description of clinical features of patients by etiology of sARF

Clinical feature	Total (n = 240)	Prerenal (n = 36)	Renal (n = 203)	*p* value
Median age (IQR; years)	66 (53–74)	60 (48–71)	67 (54–75)	0.08
Male sex (%)	139 (58)	17 (47)	122 (60)	NS
Mean Charlson co-morbidity index (±SD)	6.2 (3.6)	6.7 (3.9)	6.1 (3.6)	NS
Mean APACHE II score (±SD)	33 (8.6)	33 (7.0)	33 (8.9)	NS
Oliguria (%)	183 (77)	33 (92)	150 (74)	0.02
Hypotension (%)	204 (85)	27 (75)	177 (87)	0.07
Vasopressors (%)	185 (77)	25 (69)	160 (79)	NS
Shock (%)	178 (74)	23 (64)	155 (76)	NS
Sepsis syndrome (%)	167 (70)	23 (64)	144 (71)	NS
Bloodstream infection (%)	50 (21)	5 (14)	45 (22)	NS
Mean arterial pH (±SD)^a^	7.26 (0.14)	7.18 (0.15)	7.27 (0.13)	<0.001
Mean serum potassium (±SD) (mmol/l)^a^	4.8 (1.1)	4.9 (1.3)	4.7 (1.1)	NS
Median serum creatinine (IQR; μmol/l)^a^	405 (265–515)	357 (247–514)	413 (269–517)	NS
Median serum urea (IQR; mmol/l)^a^	24 (16–33)	24 (16–33)	25 (16–33)	NS
Mechanical ventilation (%)	174 (73)	19 (53)	155 (76)	<0.01
Acute respiratory distress syndrome (%)	91 (41)	14 (39)	85 (42)	NS
Cardiac arrest (%)	43 (18)	6 (17)	37 (18)	NS

^a^Laboratory values determined prior to initiation of RRT. APACHE, Acute Physiology and Chronic Health Evaluation; IQR, interquartile range; sARF, severe acute renal failure; SD, standard deviation; NS, non-significant.

**Table 3:** Univariate analysis of categorical factors associated with death at 1 year among sARF patients

Factor	Fatality rate with factor	Fatality rate without factor	Relative risk (95% CI)	*p* value
Surgical admission	48/87	105/153	0.80 (0.65–1.0)	0.05
Cancer diagnosis	32/38	121/202	1.4 (1.2–1.7)	0.005
Diabetes mellitus	38/72	115/168	1.3 (1.0–1.7)	0.02
Liver disease	44/50	109/190	1.5 (1.3–1.8)	<0.0001
Need for vasopressors	129/186	24/54	1.6 (1.1–2.1)	0.001
RRT modality				
Continuous renal replacement	117/147	36/93	2.1 (1.6–2.7)	<0.0001
Intermittent hemodialysis	14/48	139/192	0.4 (0.3–0.6)	<0.0001
Both	22/45	131/195	0.7 (0.5–1.0)	0.02
Hypotension	140/205	13/35	1.8 (1.2–2.9)	<0.0001
Shock	125/179	28/61	1.5 (1.1–2.0)	0.001
Mechanical ventilation	119/175	34/65	1.3 (1.0–1.7)	0.03
Acute respiratory distress syndrome	75/99	78/141	1.4 (1.1–1.6)	0.003
Sepsis syndrome	118/167	35/73	1.5 (1.1–1.9)	<0.001
Septic shock	104/139	49/101	1.5 (1.2–1.9)	<0.0001

CI, confidence interval; RRT, renal replacement; sARF, severe acute renal failure.

**Table 4:** Univariate analysis of continuous factors associated with death at 1 year among sARF patients

Factor	Alive (n = 87)	Dead (n = 153)	*p* value
Median age (IQR; years)	62.5 (48–72)	71 (59–79)	<0.0001
Mean Charlson co-morbidity index score (±SD)	5.3 (± 3.6)	6.7 (± 3.5)	0.002
Mean APACHE II score (± SD)	30.3 (± 8.5)	34.8 (± 8.3)	0.0001
Median pre-dialysis creatinine (μmol/l) (IQR)	470 (341–582)	344 (250–475)	0.0001
Median pre-dialysis platelets (×10^9^/l; IQR)	156 (71–241)	83 (41–170)	0.0001

APACHE, Acute Physiology and Chronic Health Evaluation; IQR, interquartile range; sARF, severe acute renal failure; SD, standard deviation.

**Table 5:** Logistic regression model of independent factors for 1 year mortality in patients with sARF

Factor	Odds ratio (95% CI)	*p* value
Charlson co-morbidity Index (per point)	1.2 (1.1–1.3)	0.002
Liver disease (present)	6.5 (2.3–18)	<0.0001
APACHE II score (per 5 points)	1.24 (1.02–1.5)	0.04
Septic shock (present)	2.1 (1.1–4.1)	0.02
Need for continuous renal replacement (present)	6.0 (3.1–11.9)	<0.0001

Area under ROC curve 0.83, Hosmer-Lemeshow goodness-of-fit (degrees of freedom (8)), *p* = 0.78. APACHE, Acute Physiology and Chronic Health Evaluation; CI, confidence interval; sARF, severe acute renal failure.
